# Systematic identification of critically ill and dying patients in primary care using the German version of the Supportive and Palliative Care Indicators Tool (SPICT-DE)

**DOI:** 10.3205/000278

**Published:** 2020-02-03

**Authors:** Kambiz Afshar, Birgitt Wiese, Nils Schneider, Gabriele Müller-Mundt

**Affiliations:** 1 Institute for General Practice, Hannover Medical School, Hannover, Germany

**Keywords:** palliative care, general practice, primary care, identification tool, SPICT

## Abstract

**Objective:** The systematic identification of patients who are at risk of deteriorating and dying is the prerequisite for the provision of palliative care (PC). This study aimed to investigate the feasibility and practicability of the German version of the Supportive and Palliative Care Indicators Tool (SPICT-DE) for the systematic identification of these patients in general practice.

**Methods:** In the beginning of 2017, twelve general practitioners (GPs; female n=6) were invited to take part in the study. GPs were asked to apply the SPICT-DE in everyday practice over a period of two months in patients with chronic progressive diseases. Six months after initial assessment, a follow-up survey revealed how the clinical situation of the initially identified patients had changed and which PC actions had been initiated by GPs. In addition, GPs gave feedback on the practicability of SPICT-DE in daily routine.

**Results:** 10 of the 12 GPs (female n=5, median age 46 years, range 38–68) participated in both the two-month assessment period and the follow-up survey. A total of 79 patients (female n=40, median age 79 years, range 44–94) was assessed with the SPICT-DE. Main diagnoses were predominately of cardio-vascular (n=28) or oncological (n=26) origin. Follow-up after six months showed that 38 patients (48%) went through at least one crisis during the course of disease and almost one third (n=26) had died. The majority of GPs (n=7) considered the SPICT-DE to be practical in daily routine and helpful in identifying patients who might benefit from PC. Seven GPs indicated that they would use the SPICT-DE as part of everyday practice.

**Conclusions:** The SPICT-DE seems to be a practical tool supporting the systematic identification of critically ill and dying patients in general practice.

## Zusammenfassung

**Ziel:** Die systematische Identifikation von Patientinnen und Patienten, bei denen eine Verschlechterung des Gesundheitszustandes und das Versterben abzusehen sind, ist die Voraussetzung für die Einleitung einer Palliativversorgung. Diese Studie untersucht die Anwendung und Praktikabilität der deutschen Version des Supportive and Palliative Care Indicators Tool (SPICT-DE) bei der systematischen Identifikation dieser Patientinnen und Patienten in der hausärztlichen Versorgungspraxis.

**Methoden:** Anfang 2017 wurden zwölf Hausärztinnen und Hausärzte (weiblich n=6) zur Teilnahme an der Studie eingeladen, um SPICT-DE in einem Zeitraum von zwei Monaten bei Patientinnen und Patienten mit chronisch progredienten Erkrankungen anzuwenden. Sechs Monate nach der ersten Anwendung wurde in einer Nacherhebung erfasst, wie sich der Gesundheitszustand dieser Patientinnen und Patienten im Verlauf geändert hat und welche palliativmedizinischen Maßnahmen durch die Hausärztinnen und Hausärzte in der Zwischenzeit eingeleitet wurden. Ergänzend haben die Hausärztinnen und Hausärzte einen Feedbackbogen zur Praktikabilität von SPICT-DE im Praxisalltag ausgefüllt.

**Ergebnisse:** Zehn der zwölf Hausärztinnen und Hausärzte (weiblich n=5, Altersmedian 46 Jahre, Spannweite 38–68) nahmen sowohl an der Anwendungsphase als auch an der Nacherhebung teil. Insgesamt 79 Patientinnen und Patienten (weiblich n=40, Altersmedian 79 Jahre, Spannweite 44–94) wurden mittels SPICT-DE beurteilt. Die Hauptdiagnosen waren vornehmlich aus dem kardiovaskulären (n=28) oder onkologischen Bereich (n=26). Die Nacherhebung sechs Monate nach der initialen Anwendung von SPICT-DE zeigte, dass bei 38 Patientinnen und Patienten (48%) mindestens ein krisenhaftes Ereignis im Krankheitsverlauf auftrat und dass fast ein Drittel (n=26) in der Zwischenzeit verstorben war. Die Mehrheit der Hausärztinnen und Hausärzte (n=7) erachteten SPICT-DE als praktikabel in der täglichen Anwendung und hilfreich bei der Identifikation von Patientinnen und Patienten, die von einer Palliativversorgung profitieren können. Sieben Hausärztinnen und Hausärzte gaben an, dass sie SPICT-DE auch zukünftig im Rahmen der hausärztlichen Versorgungspraxis nutzen würden.

**Schlussfolgerungen:** SPICT-DE scheint ein praktisches Instrument für die hausärztliche Versorgungspraxis zu sein, um schwerstkranke und sterbende Patientinnen und Patienten systematisch zu identifizieren.

## Introduction

The needs of the majority of people at the end of life (approximately 85–90%) can be met within primary palliative care (PC) provided by general practitioners (GPs) [1], [2]. A crucial step in providing adequate PC is the systematic and timely identification of patients who might benefit from PC [3]. An appropriate identification may contribute to high-quality PC and increase patients’ quality of life, avoid unnecessary hospital admissions, reduce healthcare costs and optimize the provision of primary PC by GPs [4], [5]. However, the identification of patients with potential PC needs is a challenging task, not least because of prognostic uncertainty in oncological and even more in non-oncological conditions [6], [7], [8], [9]. In Germany, the identification of patients with both oncological and non-oncological diseases who might benefit from PC is inconsistent [10], [11], [12]. No supportive tool has been systematically investigated or established for application in general practices in Germany before.

Internationally, different clinical tools have been developed and implemented to support the identification of patients with potential PC needs [13], [14], [15]. One of these tools is the Supportive and Palliative Care Indicators tool (SPICT), a clinical tool first developed in 2010 as a collaborative project between NHS Lothian and the University of Edinburgh Primary Palliative Care Research Group [16]. Studies indicate that SPICT is a helpful and practical tool to support the identification of patients who might benefit from PC in different settings [17], [18], [19], [20]. The SPICT-DE is the German version of the SPICT that has been systematically developed, refined and pretested for its application in general practices recently [21].

Nevertheless, data is missing on the acceptance and practicability of SPICT-DE in general practice in Germany. The aim of this study was to assess the feasibility of the SPICT-DE by GPs in everyday practice and to elucidate whether the SPICT-DE supports the identification of patients being at risk of deteriorating and dying in primary care.

## Methods

The study was designed as a prospective exploratory feasibility study with a two-month assessment phase (t0) and a follow-up survey after six months (t1).

### SPICT-DE

The SPICT-DE is the German version of the Supportive and Palliative Care Indicators Tool (SPICT) [21]. The systematic development, adjustment and pretesting of the SPICT-DE was completed in a multiprofessional and participatory approach in 2017 [21]. It is a single-page tool with a three-part structure comprising:

1. general clinical indicators (e.g. unplanned emergency hospital admissions or weight loss in the past six months),
2. condition-specific clinical indicators (e.g. in cancer, frailty, cardiovascular, pulmonary or liver diseases), and
3. recommendations for PC actions (e.g. a review of medication, a conversation about deteriorating health and dying with patients and their relatives, advance care planning, or referral for specialist PC).

The SPICT-DE used in this feasibility study did not include a cut-off value as it did in the version before 2017 (≥2 general indicators; version of 2014). The latest version of SPICT-DE can be downloaded free of charge from the SPICT website [22]).

### Participants

In this study, we followed a purposive sampling strategy. In the beginning of 2017, twelve GPs (female n=6) from rural and urban regions with different professional backgrounds, different working experience in general practice, with and without a further qualification in PC were invited to participate. Inclusion criterion was a working experience of at least two years in general practice. All GPs who applied the SPICT-DE in daily routine before were excluded from participation.

### Assessment period (t0)

One member of the study team (KA or GMM) visited the GPs for user training and initiating the assessment period. To ensure a common understanding of the term “palliative care”, a German definition based on the German Guideline “Palliative care for patients with incurable cancer” [23] and on the World Health Organization [24] was given to each GP in hard copy. Subsequently, a standardized user training (duration: approximately 15 minutes) was performed by the visiting member of the study team (KA or GMM) to illustrate the application of SPICT-DE according to the recommendations of the SPICT-DE Guide 2019 [22]. GPs then were asked to apply the SPICT-DE in daily practice over a period of two months. This period was chosen in order to minimize the time burden for the GPs and to promote collaboration. The SPICT-DE should be applied in any patient that would visit the practice or would be seen in domiciliary visit regardless of their place of living (e.g. at home, nursing home or care facility) during the assessment period and that would meet the following inclusion criteria: age≥18 years with at least one oncological or non-oncological chronic life limiting disease according to the SPICT-DE. Patients who had previously been referred to specialized PC or who were residents of hospices were excluded.

For each patient meeting the inclusion criteria, GPs were asked to highlight all applicable indicators of the SPICT-DE. In order to monitor the indicators chosen by the GPs, check boxes were added for each indicator and recommended PC action listed in the SPICT-DE. There was the opportunity to mention any additional actions as a free-text answer as well.

### Supplementary questionnaire

In order to gather additional information and to evaluate the handling of SPICT-DE in daily practice, we designed a supplementary questionnaire. This semi-structured questionnaire consisted of three parts. The first part included five questions on the practice structure and on GPs’ sociodemographic as well as professional data. The second part included six questions and was designed to acquire further information on each patient assessed with the SPICT-DE. Beside patients’ sociodemographic data, GPs were asked to give further information on the underlying main disease(s) and therapies, on existing PC, care services and need of long-term care, and on the existence of a patient will and advance directives. The third part consisted of six questions to obtain feedback on the practicability of the SPICT-DE in everyday practice and to survey if the SPICT-DE was considered helpful in identifying patients who might benefit from PC. This questionnaire was also designed to identify a potential need for adjustment of the SPICT-DE for its application in general practice. Furthermore, GPs were asked to state if using SPICT-DE altered their view on PC patients in general and if they could imagine using SPICT-DE further in daily practice. Each question provided the opportunity to give free-text answers as well.

### Follow-up (t1)

Six months after the initial application of the SPICT-DE in general practice, all GPs gave informed consent to participate in a follow-up survey to reveal whether and how the clinical situation of the patients had changed. Therefore, GPs were asked to fill out a single-page semi-structured questionnaire with six questions concerning alterations in the patients’ situation and the occurrence of any critical incidences for every patient identified with SPICT-DE during the past six months. A critical incidence was defined as acute crises in the disease progression, unplanned hospital admissions, changes in therapy, care, and living environment as well as death of patients. Furthermore, GPs were asked to indicate for each patient if – and if so, which – PC actions as recommended by the SPICT-DE had been initiated in the meantime. GPs did not know from the beginning that they would take part in the follow-up survey in order to avoid effects on the assessment period (t0).

### Ethical approval and data protection

The study was approved by the ethics committee of Hannover Medical School in December 2014 (No.: 2499-2014). All GPs in this study gave informed consent prior to participation. Each GP was assigned with an individual code in order to pseudonymise GP-related data. The code list was archived separately from the data collection documents. GPs listed each patient assessed with SPICT-DE and assigned them with an individual ID. That list remained in the practices and was inaccessible for the study team. All patient data collected and stated by the GPs in the questionnaires were given completely anonymously to the study team so that patients’ identity was fully preserved at any time.

### Data analysis

As mixed methods were applied, quantitative and qualitative analyses were performed. Quantitative data were analysed with descriptive statistics for small samples using the Statistical Package for the Social Sciences (SPSS) Version 25.0. Spearman correlation was used to evaluate the relationship between the total number of indicators documented at baseline (t0) and the number of critical incidences occurred during the period of observation (t1). Responses to the open-ended questions from the supplementary questionnaire were analysed by conventional content analysis as described by Hsieh and Shannon [25].

## Results

### Participants

From the twelve GPs initially invited to take part in this study, two GPs declined to participate due to lack of time, and ten gave informed consent to participate. These ten GPs came from nine different general practices in Lower Saxony and North Rhine-Westphalia and participated in both the two-month assessment period and the follow-up survey six months later. The sample consisted of five women and five men with a median age of 46 years (range 38–68 years). Four GPs had a further qualification in PC. GPs stated a median of 1,223 (range 900–2,950) patient consultations in one quarter of the year. GPs’ characteristics and sociodemographic data are shown in Table 1 (Tab. 1).

### Application of the SPICT-DE

Over the investigation period, the SPICT-DE was applied in 79 patients (female n=40, median age 79 years, range 44–94 years) who met the inclusion criteria. The number of patients per GP for whom the SPICT-DE was applied ranged from 3 to 15 (median 8). Patient characteristics are summarised in Table 2 (Tab. 2).

### Indicators of the SPICT-DE

General indicators of the SPICT-DE were applicable in 73 patients. The median number of applicable general indicators was 4 (range 0–7). The three most frequently applied indicators were *“Depends on others for care due to increasing physical and/or mental health problems”* (n=60), *“Performance status is poor or deteriorating, with limited reversibility (e.g. The person stays in bed or in a chair for more than half the day)”* (n=59), *“Persistent symptoms despite optimal treatment of underlying condition(s)”* (n=40), and *“The person’s carer needs more help and support” *(n=40). Specific indicators of the SPICT-DE were applicable in 76 patients. The median number of applicable specific indicators was 1 (range 0–4). Heart and vascular diseases (n=28), cancer (n=26) and dementia/frailty (n=23) were the most often mentioned specific diseases according to the SPICT-DE. Table 3 (Tab. 3) gives an overview of all applicable indicators of the SPICT-DE.

### PC actions

PC actions as recommended by the SPICT-DE were considered suitable in all 79 patients (Table 4 (Tab. 4)). The median number of applicable PC actions was 3 (range 1–6). The most frequently mentioned actions of the SPICT-DE were *“Agree a current and future care plan with the person and their family; support family carers”* (n=59), *“Review current treatment and medication to ensure the person receives optimal care; minimise polypharmacy”* (n=53), and *“Plan ahead early if loss of decision-making capacity is likely”* (n=49).

### Follow-up

Six months after the initial application of the SPICT-DE, follow-up showed that 38 patients (48%) went through at least one critical incident in the disease progression: acute crisis, hospital admission, altered care needs and death (Table 5 (Tab. 5)). At the time of follow-up, almost one third of the patients (n=26) had died. There was a low but statistical significant correlation between the total number of indicators documented at baseline (t0) and the number of critical incidences that occurred during the period of observation (t1) (Spearman r=0.253; p=0.024).

GPs had initiated several PC actions for the patients identified with SPICT-DE in the meantime (median 1, range 0–7). The most frequently initiated PC actions were to review the current medication and treatment (n=36), advance care planning (n=29), and referral for specialist assessment due to complex symptoms or problems (n=24) (Table 4 (Tab. 4)). There was an average correlation (Spearman r=0.465) between the number of consideration (t0) and the number of corresponding actual initiations (t1) of PC actions as recommended by the SPICT-DE (p<0.001). 

### Feedback on the application of SPICT-DE

The majority of GPs (n=7) shared the opinion that SPICT-DE is helpful in identifying patients who might benefit from PC. Seven GPs considered the application of the SPICT-DE to be practical in everyday practice to identify patients who might benefit from PC. Four GPs stated that the application of SPICT-DE changed the consideration of PC patients: One GP elaborated that SPICT-DE sharpens his view. Two GPs explained that SPICT-DE helped to consider also patients with non-oncological diseases for PC and to remember talking about advance care planning. One GP stated that psychosocial aspects would not find sufficient consideration in the SPICT-DE. Only two GPs considered SPICT-DE to be too complex and time-consuming. Seven GPs indicated that they could imagine using SPICT-DE further as part of their daily routine. One GP indicated to be still indecisive. There were no significant differences in the GPs’ answers according to sex, age, professional experience and specialisation.

## Discussion

This prospective exploratory feasibility study indicates that SPICT-DE seems to be a practical tool in general practice to identify patients who might benefit from PC and to support the initiation of PC actions.

While the first testing of SPICT-DE on an internal ward in a hospital revealed a need for adjustment in language and layout in particular [19], results of this study indicate that SPICT-DE is feasible, comprehensible and practicable in everyday general practice. Besides, the majority of GPs emphasized the simple handling of SPICT-DE in their feedback. Furthermore, the SPICT-DE seems to include the most important indicators and dimensions for an adequate identification of patients with potential PC needs. GPs did not state that any essential indicators were missing to them. These findings are in line with the results of the systematic development and testing of SPICT-DE in quality circles with German GPs [21].

In line with previous studies [16], [17], the results of this study also suggest that the SPICT-DE identifies patients with a dynamic disease progression and who are at risk of deteriorating or dying within the near future. About one third of all patients in this study died within six months after the identification using the SPICT-DE. Applying the SPICT-DE in regular intervals might support the perception for changes in the patient’s situation and increase the awareness of GPs for a timely initiation of PC actions.

It is remarkable that GPs considered the PC action *“Agree a current and future care plan with the person and their family; support family carers”* to be indicated in 75% of the patients (n=59). The follow-up survey revealed that this PC action was effectively initiated in only ten patients. Similar results are shown for the indicator *“Record, communicate and coordinate the care plan”* (t0: n=39 vs. t1: n=8). In contrast, the PC action *“Consider referral for specialist assessment if symptoms or problems are complex and difficult to manage”* was actually initiated in 23/25 patients. The identification of patients in potential need of PC is a crucial step in the provision of PC. Subsequent actions including a conversation on end-of-life issues with the patients identified require an ethically sensitive approach. Initiating a conversation on end-of-life issues is not an easy task requiring special communication skills and professional experience [26]. As a beneficial precondition, patients and their relatives need to have an open mind when initiating a conversation on end-of-life issues, especially at an early stage of disease trajectory [27]. In these cases, GPs play a key role in sensitively promoting the receptiveness of patients and their relatives for advance care planning [28].

Another important aspect is that the follow-up survey revealed first information on the effects of applying SPICT-DE in daily practice. The consideration and the actual initiation of PC actions as recommended by the SPICT-DE correlated significantly, although GPs did not know from the beginning that they would take part in the follow-up survey. This indicates a general acceptance of the SPICT-DE and an intrinsic motivation for using SPICT-DE in daily routine. The SPICT-DE was considered helpful by GPs regardless of the professional background, practice structure, amount of PC patients in total or qualification in PC.

The SPICT does not include a cut-off value of deteriorating health anymore as it was part of older versions before 2017 (≥2 general indicators; version of 2014). Recent research on SPICT from Belgium and Japan favours using a cut-off value [17], [18]. Nevertheless, GPs in this study did not indicate that they would miss a cut-off value to use the SPICT-DE properly. This may be linked to the user training performed to illustrate the application of SPICT-DE according to the recommendations of the SPICT-DE Guide 2019 [22]. GPs used SPICT-DE as the originators recommend it: as a clinical decision-making aid or an aide memoire.

### Strengths and limitations

This is the first feasibility study evaluating the application of the SPICT-DE in daily routine by GPs in Germany. The combination of the assessment period with a follow-up survey six months later increased the conclusiveness of the results. The follow-up survey provides important data on the sensitivity of the SPICT-DE in identifying patients with complex needs who might benefit from the initiation of PC.

A small and selective sample of ten GPs participated in this study. The majority of GPs belonged to teaching and research practices of the Institute for General Practice. Thus, it can be assumed that these GPs might have a higher motivation to take part in research and to integrate new tools in practice compared to other GPs. An interventional study with a more representative and a larger sample of GPs is needed.

Data of overall 79 patients of 10 GPs were available for statistical analysis. Considering the sample size, further statistical analysis and comparison of subgroups were not possible or rather not reasonable.

The initiation of PC actions was not only assessed for those patients for whom initiation was considered at t0. It is possible that there might be patients with PC action initiation at t1 without a documented consideration at t0 and vice versa.

## Conclusions

The SPICT-DE seems to be a practical clinical tool supporting the systematic identification of patients with potential PC needs who are at risk of deteriorating or dying. The results of this study suggest that the application of the SPICT-DE seems to be feasible in general practice. The SPICT-DE might increase GPs’ awareness for patients with potential PC needs and contribute to initiating PC actions for patients with different chronical progressive diseases. Its indicators correlate significantly with the occurrence of a critical incidence in the patient’s situation within six months. The results of this study are a prerequisite for a following intervention study to evaluate the implementation of SPICT-DE in routine daily practice [29]. Further research will elucidate if the systematic application of SPICT-DE in general practice results in an optimisation of care for patients with chronic progressive diseases at the end of life.

## Notes

### Competing interests

The authors declare that they have no competing interests.

### Funding

Funding for this research was provided by the internal research fund of Hannover Medical School (Fonds-No.: 79273002).

### Acknowledgements

We thank all general practitioners who participated in this study. We also thank Daniela Werth and Fabian Tetzlaff for supporting the data preparation.

## Figures and Tables

**Table 1:**
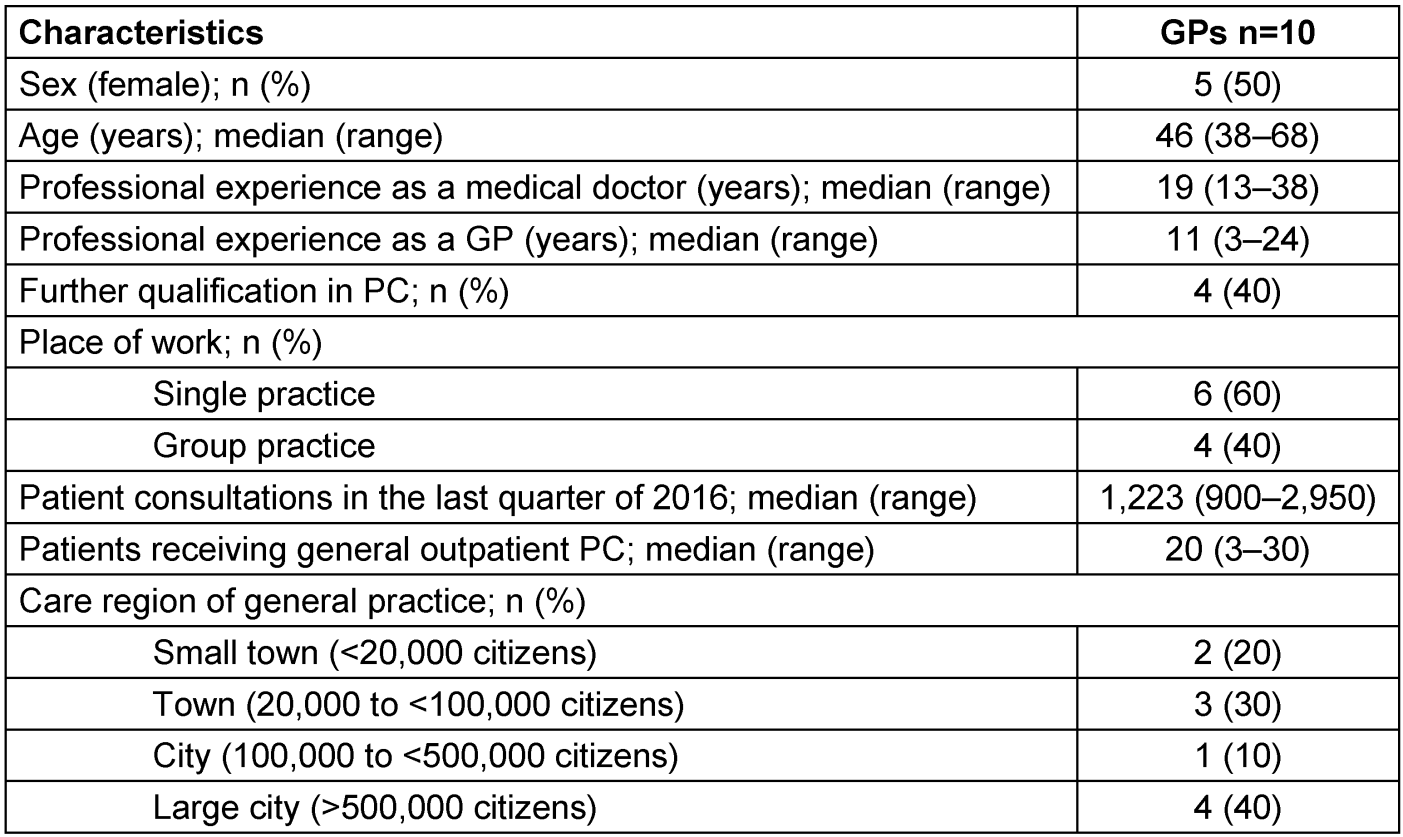
Characteristics and sociodemographic data of participating GPs (n=10)

**Table 2:**
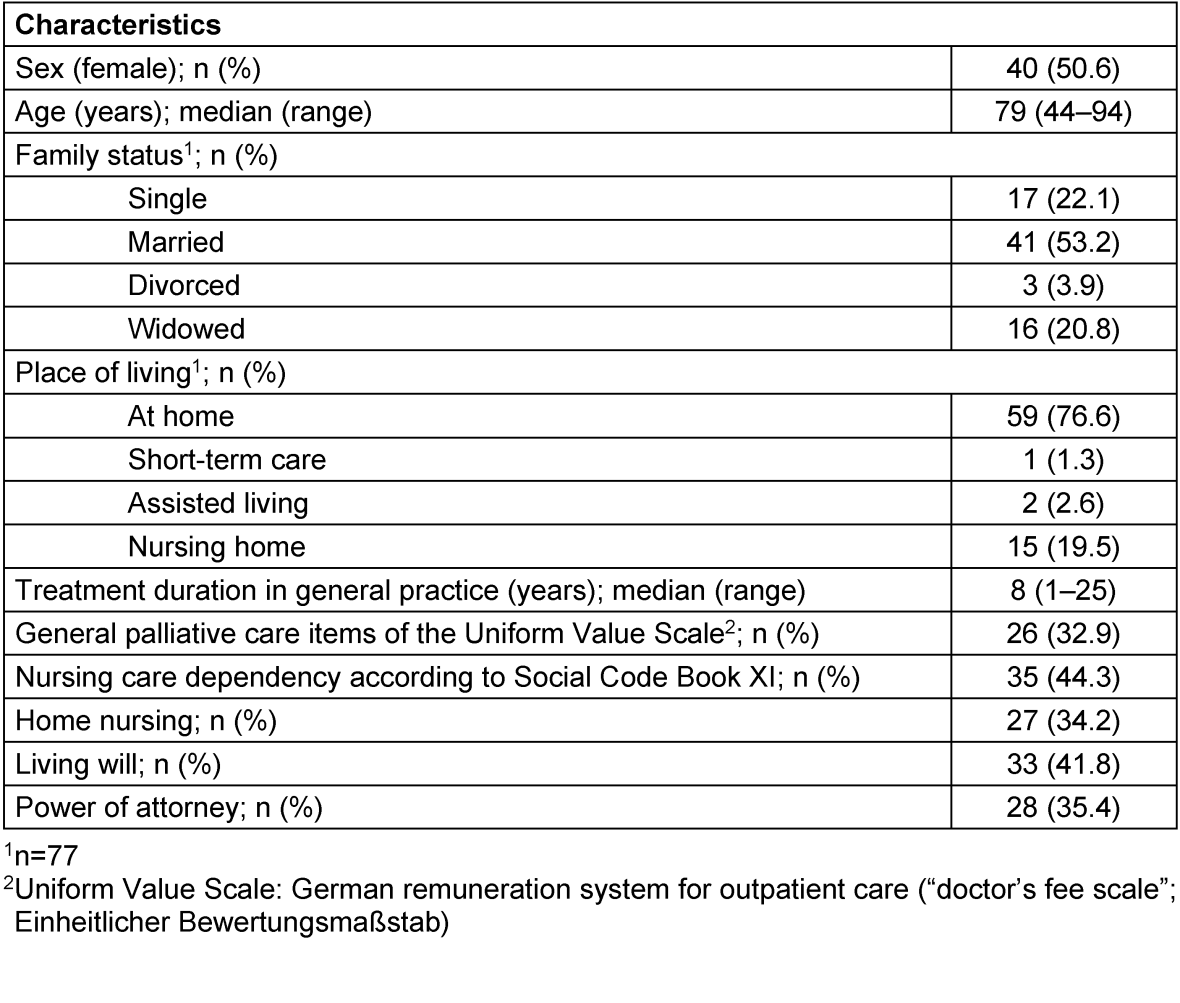
Characteristics and sociodemographic data of patients assessed with the SPICT-DE

**Table 3:**
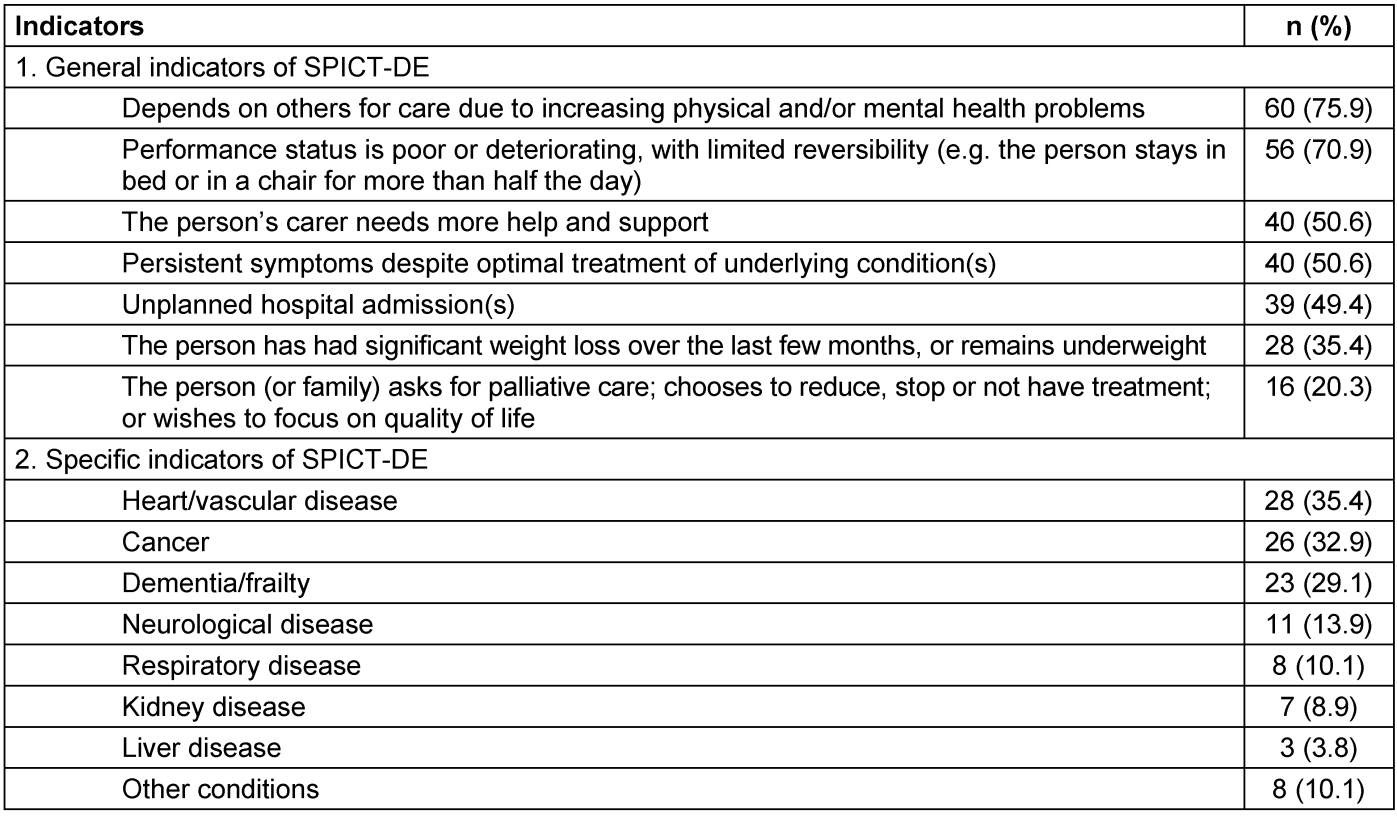
General and specific indicators of the SPICT-DE applicable for patients during the assessment period (n=79; multiple responses allowed)

**Table 4:**
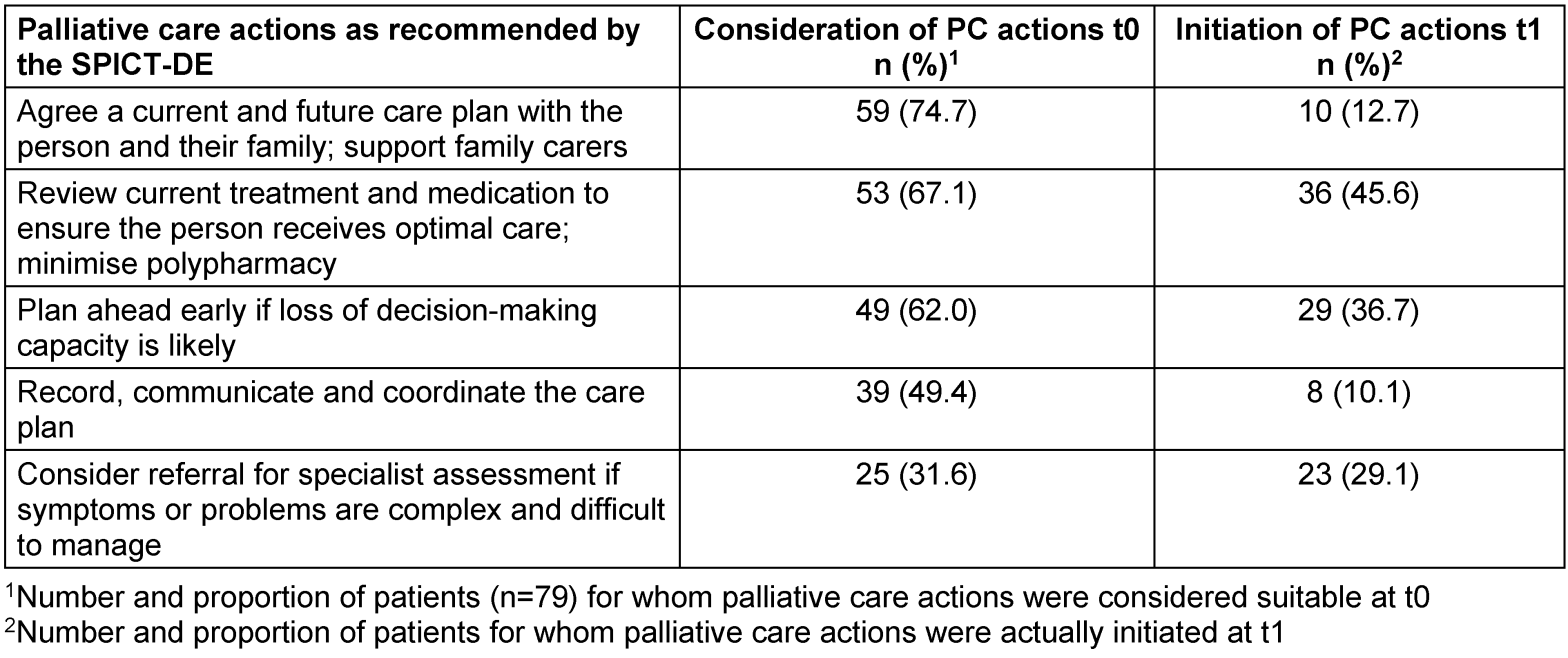
Comparison of the consideration (t0) and initiation (t1) of PC actions as recommended by the SPICT-DE (n=79; multiple responses allowed)

**Table 5:**
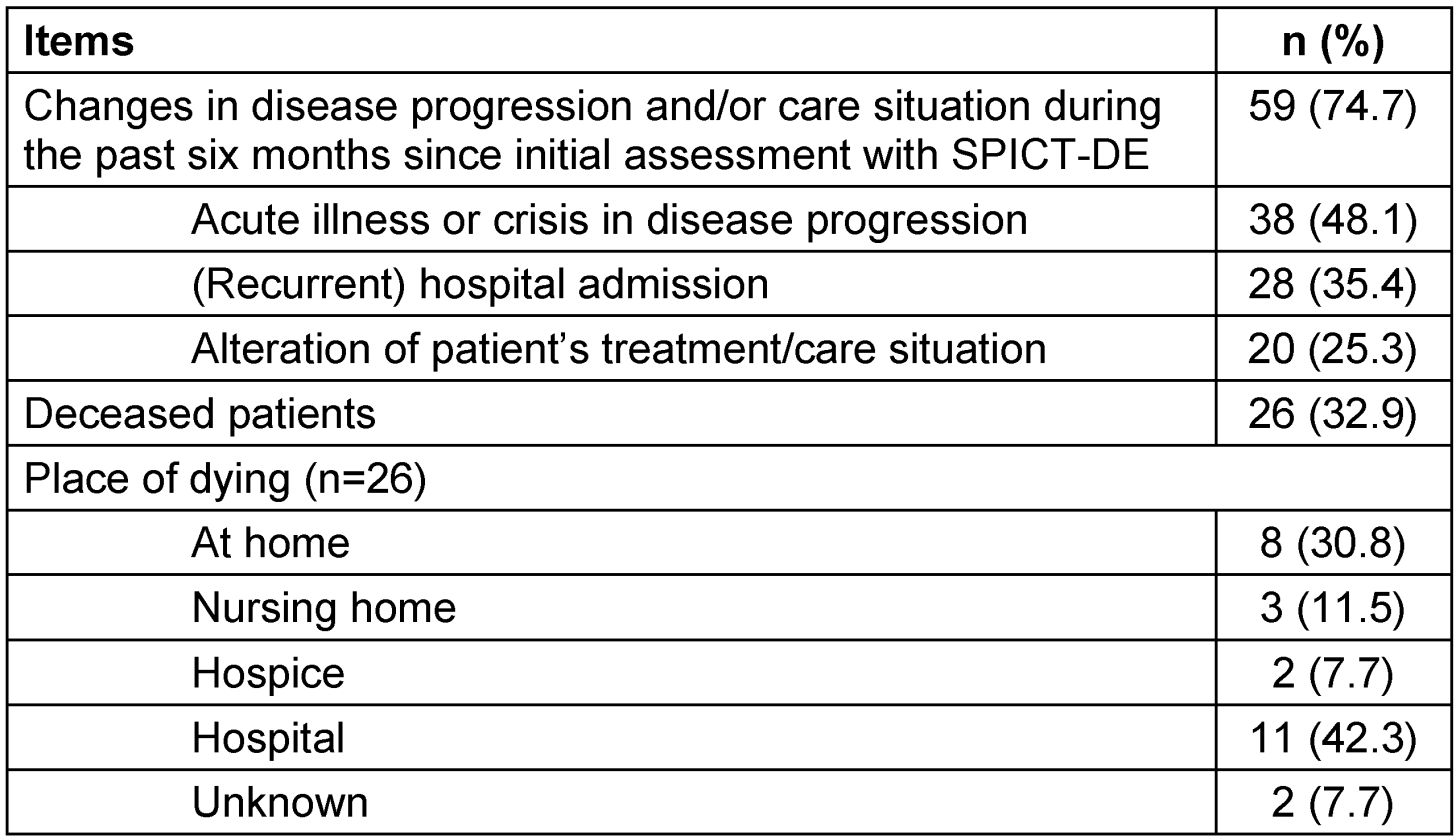
Follow-up results six months after initial assessment with SPICT-DE (n=79)
